# Supplementary material for: Incidence of major depressive disorder over time in patients with liver cirrhosis: A nationwide population-based study in Korea
Source: PLoS One. 2022 Dec 9;17(12):e0278924. doi: 10.1371/journal.pone.0278924 (PMC9733842; doi:10.1371/journal.pone.0278924)
Supplement: Attachment — Submitted filename: Comment PLOS 2Oct22.docx [file pone.0278924.s001.docx]

Oct 2, 2022

Dear Authors submitting to Journal PLOS ONE

**Manuscript Title:** Incidence of major depressive disorder over time in patients with liver cirrhosis: A nationwide population-based study in Korea

**Manuscript ID:** PONE-D-22-24164

**All Authors list:** Gi Hyeon Seo, Jeong-Ju Yoo

**Manuscript Type:** Research Article

1. The purpose of the article was to examine the incidence of major depressive disorder (MDD) among patients with cirrhosis from the time before and after their diagnosis, using a retrospective analysis of the ICD-10.
2. There was a very interesting study, and I have the following concerns to address: kindly be informed that the author provided details regarding the materials and techniques, including the methodology, flow diaphragm of study enrollment using inclusion and exclusion criteria, as well as statistical analysis used in the article.
3. Population: Due to the article's focus on the incidence of major depressive disorder (MDD) in cirrhotic patients both before and after diagnosis, the precise moment of each disease's diagnosis had a significant impact on the study's findings. How does the author use the ICD-10's first-ever documentation of "cirrhosis" and "depression"? In the methodology, please be more specific.
4. What does "an incorrect mortality code" mean in the context of the materials and procedures on the 48th line?
5. Would the author kindly clarify on how they classified the time before and after the cirrhosis diagnosis using eight periods?
6. Following the result section on lines 78 to 82 of the paragraph as follows: “possibly due to the aggravation of underlying condition before cirrhosis (e.g. chronic hepatitis) that gradually  deteriorated patients’ mental health.” Are there any supporting facts or just the author's opinions? Please reconsider the phase change or switch to the discussion part.
7. According to the author mention in the discussion part: "psychological trauma immediately after diagnosis might be the most influential factor." From the design of the retrospective study, there was overemphasis. Kindly ask the author to reconsider.
8. The author mentions the following in the discussion section: “because of the high basal incidence, the relative risks were lower in  females and elderly patients than in males and younger patients.” According to another statement, "which even increased after the start of liver cirrhosis," it was difficult for the reader to understand and may have been misunderstood.
9. Additionally, the majority of illnesses that have an impact on mental health or depression are chronic illnesses such chronic liver disease or cancer, including hepatocellular carcinoma. Some cirrhotic patients, particularly those with a Child-Pugh score of A or treatable illnesses like hepatitis C infection, may not experience symptoms of their condition. This article lacked important information.
10. Last but not least, the disease's treatment for both cirrhosis and depression affects the incidence in each stage of the illness, such as medically controlling for depression, curing hepatitis C disease, and fibrosis regression in NASH. Once more, this articel was lacking crucial details.
11. Minor comment: Figure 1's right column contains an incorrect term for the “pre-diagnosis stage” of cirrhosis. Kindly ask the author to reconsider.

**In summary - my opinion: Rejection due to several limitations**

**Best regards,**

**Thaninee Prasoppokakorn,**

Division of Gastroenterology, Department of Medicine, Faculty of Medicine, Chulalongkorn University and King Chulalongkorn Memorial Hospital, Thai Red Cross Society, Bangkok, Thailand
