# Supplementary material for: Incidence of major depressive disorder over time in patients with liver cirrhosis: A nationwide population-based study in Korea
Source: PLoS One. 2022 Dec 9;17(12):e0278924. doi: 10.1371/journal.pone.0278924 (PMC9733842; doi:10.1371/journal.pone.0278924)
Supplement: Attachment — Submitted filename: Response_to_Reviewers_Yoo_et_al_20221111.docx [file pone.0278924.s003.docx]

**Responses to the Reviewers’ Comments**

11 November, 2022

Dear reviewers and editorial staff of *PLOS ONE*

We extend our sincere gratitude for your thorough consideration and scrutiny of our manuscript, “**Incidence of major depressive disorder over time in patients with liver cirrhosis: A nationwide population-based study in Korea**”, control number PONE-D-22-24164R1. The accurate comments of the reviewers have helped us to better understand the critical issues of this paper. We have revised the manuscript according to the reviewers’ suggestions. We hope that our revised manuscript will be considered and accepted for publication in *PLOS ONE*. We acknowledge that the scientific and clinical quality of our manuscript was improved by the scrutinizing efforts of the reviewers and editors.

The changes made within the revised manuscript were highlighted (underlined and in blue). Point-by-point responses to the reviewers’ comments are provided below.

**Reviewer #1 :**

***<MAJOR COMMENTS>***

**1) Reviewer’s comment:** Under Abstract and Results, the IRR was reported but no p value was given. Kindly include the p values for the relevant IRRs in the Abstract and Results.

**Author’s response:** We appreciate the reviewer’s insightful comment. We added p values in the abstract and result section.

**2) Reviewer’s comment:** Under Abstract, it will also be useful to the reader for authors to define the control period as 1 to 2 years before diagnosis of cirrhosis.

**Author’s response:** We thank the reviewer for enhancing the quality of our manuscript. According to the reviewer's opinion, definition of control period was added to the abstract.

**3) Reviewer’s comment:** Lastly, this is pertaining to the Reviewer #1, Minor Comments 3 on rising incidence rate of depression 1 year before diagnosis compared to control period of 1 to 2 years before diagnosis of cirrhosis. Could the authors consider amending lines 1 to 2 of paragraph 4 under Discussion as "Another notable point in our study is that the incidence of MDD is increasing 1 year before compared to the control period of 1 to 2 years before the diagnosis of cirrhosis".

**Author’s response:** We strongly agree with the reviewer’s opinion. We modified the sentence as the reviewer’s recommendation.
